# Independent and joint associations of sedentary behaviour and physical activity with risk of recurrent cardiovascular events in 40,156 Australian adults with coronary heart disease

**DOI:** 10.1016/j.ajpc.2025.100998

**Published:** 2025-04-17

**Authors:** Amanda Lönn, Suzanne J Carroll, Theo Niyonsenga, Adrian Bauman, Rachel Davey, Robyn Gallagher, Nicole Freene

**Affiliations:** a Department of Physical Activity and Health, The Swedish School of Sport and Health Sciences (GIH), Stockholm, Sweden; b Health Research Institute, University of Canberra, Bruce, ACT, Australia; c Sydney School of Public Health, The University of Sydney, NSW 2006, Australia; d Charles Perkins Centre, Susan Wakil School of Nursing and Midwifery, Sydney Nursing School, Faculty of Medicine and Health, The University of Sydney, NSW 2600, Australia

**Keywords:** Ischemic heart disease, Major adverse cardiovascular event, Exercise, Sedentary time

## Abstract

**Objective:**

Explore the independent and joint associations between sedentary behaviour and physical activity with cardiovascular events, among individuals with coronary heart disease (CHD).

**Methods:**

Cohort study including Australians ≥45 years with CHD (2006–2020). Time in sedentary behaviour, walking, moderate-, and vigorous- physical activity were self-reported. Cardiovascular events were identified using health registers (2006–2022). Cox proportional hazard regressions explored the association. Restricted cubic splines explored the shape of the association.

**Results:**

There were 40,156 individuals included, with a mean age of 70 (SD=10) years old, 62 % men. During a median of 8.3 (IQR = 10.03) years, 3260 non-fatal-, 5161 total cardiac events, and 14,383 major adverse cardiovascular events (MACE) were recorded. Sedentary behaviour of 7–10.4 h/day was associated with a 15 % lower risk of total cardiac events and MACE compared to ≥ 10.5 h/day. A higher level of moderate-to-vigorous physical activity was associated with a lower risk of cardiovascular events, with 14–21 % lower risk for 1–149 min/week compared to 0 min/week. A similar pattern was seen for walking and activities at a moderate- or vigorous intensity. The joint association of ≥150 min/week of moderate-to-vigorous physical activity and <7 h/day in sedentary behaviour had the lowest risk (29–48 % lower) for cardiovascular events compared to the reference group. However, moderate-to-vigorous physical activity seems to be of greater importance and partly modifies the risk of sedentary behaviour in the joint association. Sedentary behaviour hours were linearly associated with risks of non-fatal and total cardiac events. Meanwhile time in physical activity had a curvilinear association with cardiovascular events, with the greatest benefits at the beginning of the curve.

**Conclusion:**

More time in physical activity and less time in sedentary behaviour are associated with a lower risk of cardiovascular events. This emphasizes the importance of providing recommendations for both physical activity and sedentary behaviour to people with CHD.

## Highlights

• Any physical activity is associated with a lower risk of cardiovascular (CVD) events.
• Physical activity benefits CVD most at the start, with a curvilinear association.
• Low level of sedentary behaviour is associated with lower risk of severe CVD event.
• Sedentary behavior linearly associated with CVD, no max effect with less time.
• Best effect with high physical activity and low sedentary behavior combined.

## 1 Introduction

Cardiovascular disease is globally widespread of which coronary heart disease (CHD) represents a high proportion [1]. The acute treatment of CHD has improved in recent decades, contributing to greater survival following the acute phase [2]. However, the risk of recurrent events is high with ∼30 % having a readmission related to cardiovascular causes within two years after a myocardial infarction [3], emphasizing the need for secondary prevention. Physical activity has been recommended in secondary prevention, due to its positive association with cardiovascular risk factors [[4], [5], [6]], and mortality [7,8]. There is no consensus on the association of physical activity with recurrent non-fatal cardiac events. One large multicentre study among individuals with CHD found no difference in the risk of recurrent myocardial infarction or stroke among the highest tertile of physical activity compared with the lowest [8]. Meanwhile, another study found a 25 % lower risk of cardiovascular-related readmissions among individuals reporting ≥ 60 min/week compared with inactive individuals [9]. The association between time in sedentary behaviour and the risk of recurrent cardiovascular disease events has been less well explored. A small study among patients with heart disease found a 42 % lower risk of total hospital readmission and 56 % lower risk of all-cause mortality among individuals reporting ≤ 6 h of sedentary behaviour compared to ≥ 10 h/day [9].

Additionally, the shape of the association between time physical activity and the risk of recurrent cardiovascular events among individuals with CHD is scarcely explored. Among individuals with cardiovascular disease, the association is curvilinear between time in physical activity and risk of cardiovascular and all-cause mortality, with a steep initial slope and the greatest reduction in risk at the beginning of the curve [[10], [11], [12]]. Two previous studies have explored the shape of the association with recurrent cardiovascular events among individuals with cardiovascular disease, with conflicting results. Bonekamp et all found a curvilinear association between the volume of leisure physical activity and the risk of a cardiovascular event [10]. Meanwhile, Bakker et al. found that the shape of the association between time in physical activity with major adverse cardiovascular events (MACE) was linear, indicating no maximal effect of physical activity [13]. To our knowledge, no previous studies have explored the shape of the association between time in sedentary behaviour with the risk of morbidity or mortality among individuals with any cardiovascular disease.

At present there are no diagnosis-specific guidelines for the time in sedentary behaviour and physical activity for individuals with CHD. Additionally, the joint association of physical activity and sedentary behaviour with cardiovascular events for individuals with CHD has not been previously explored. Thus, this study aims to explore the independent and joint associations between time in sedentary behavior and physical activity with recurrent cardiovascular events (non-fatal cardiac events, total cardiac, and MACE) in a cohort with CHD. Further, this study explores the shape of the curve for the association between time in sedentary time and physical activity and the risk of recurrent cardiovascular events among individuals with CHD.

## 2 Methods

This study is based on individuals from the Australian ‘45 and up’ prospective cohort study. This cohort consists of 267,151 individuals ≥ 45 years, randomly sampled from the general population in New South Wales, Australia. Individuals were included by providing written informed consent and answering the questionnaire [14]. Survey data were collected in waves; first (2006 to 2009), Social, Economic and Environmental Factors (SEEF) (2010), second (2012–2016), and third (2018–2020). The NSW Population and Health Services Research Ethics Committee approved this study (2022/ETH00559).

To be included in this study, individuals self-report they had CHD. This was assessed by the following questions, *being treated for a ‘heart attack or angina’ or ‘other heart disease’ in the last month* or ‘*ever told heart disease as diagnosed by a doctor’*, or *‘having a coronary artery bypass graft operation’*. Individual's baseline was defined as the date when they first stated in a questionnaire that they had been diagnosed with CHD.

### 2.1 Outcomes

To identify the first recurrent cardiovascular disease event, the dataset was linked with the Admitted Patient Data Collection (APDC), New South Wales [15] and the New South Wales Register of Births, Deaths & Marriages – Death Registrations and the Australian Bureau of Statistics Mortality Data [16]. International Classification of Diseases (ICD-10) codes were used to identify: (i) non-fatal cardiac events (I21-I24 using the APDC dataset); (ii) total cardiac events including non-fatal and fatal events ((i) plus I20–125 and I46.2 using the mortality datasets); and (iii) MACE including total cardiac events, stroke, heart failure and cardiovascular death ((ii) plus I63, I65, I66, I11.0, I50 and I97 using the APDC dataset, and I00-I99, I46.1 and I46.9 using the mortality datasets) [17]. Recurrent CVD events occurring within 90 days were excluded as they could be related to the previous event. The participants were followed until they had their first recurrent cardiovascular disease event or end date of the study (31–12–2022).

### 2.2 Exposures

The questions for sedentary behaviour were, ‘*how many hours in each 24-hour day they spent: sitting; watching TV/using a computer’*, in the first and SEEF waves. In waves 2 and 3 individuals were asked *how much time they spent in the last 7 days on a usual weekday and weekend day sitting for: transport; work; watching TV; using computer at home; other leisure activities* (Supplementary 1). The average daily time in total sedentary behaviour was calculated using (weekday total sedentary behaviour x 5 + weekend total sedentary behaviour x 2)/7. If participants responded to at least one sedentary behaviour question, 0 min were recorded for missing data fields. The maximum cut-off of sedentary behaviour was set to 16 h per day. Time in sedentary behaviour was handled as continuous, quartiles, and categories. The categories were divided into 0–3.4 h/day, 3.5–6.9 h/day, 7–10.4 h/day, and ≥10.5 h/day.

Physical activity in the last week was measured using the Active Australia Survey [18], which has demonstrated an acceptable level of agreement for time in moderate-to-vigorous physical activity compared to accelerometer measurement among patients with CHD [19]. The survey consists of questions about walking, moderate-intensity physical activity, and vigorous-intensity physical activity (Supplementary 1). For walking, moderate-intensity physical activity, and vigorous-intensity physical activity, the maximum cut-off was set to 840 min per week [18]. Total time in moderate-to-vigorous physical activity (MVPA) was calculated using the standardized calculation: walking + moderate-intensity physical activity + (2 x vigorous-intensity physical activity) [18]. The maximum cut-off in total MVPA was set to 1680 min per week. If the individual responded to at least one physical activity question, 0 min were assigned for the other physical activity questions if the data were missing. Time in physical activity was handled as continuous, quartiles, and categories. The categories for walking, moderate-intensity physical activity, and MVPA were 0 min/week, 1–149 min/week, 150–300 min/week, and ≥ 300 min/week. Vigorous-intensity physical activity was categorized as 0 min/week, 1–74 min/week, 75–150 min/week, and ≥150 min/week.

To explore the joint association of sedentary behaviour and MVPA, data were combined to create categories. Time in sedentary behaviour was dichotomized into < 7 h/day and ≥ 7 h per day, based on the increased risk of all-cause mortality among individuals self-reporting ≥7 h/day sedentary time [20]. MVPA was dichotomized into ≥150 min/week or < 150 min/week, based on international recommendations of physical activity to promote health for a general adult population [21]. Sedentary time and MVPA were then categorized into: MVPA <150 min/week and sedentary behaviour > 7 h/day; MVPA <150 min/week and sedentary behaviour <7 h/day; MVPA ≥150 min/week and sedentary behaviour >7 h/day and MVPA ≥150 min/week and sedentary behaviour <7 h/day.

### 2.3 Covariates

Included covariates were age, sex (male, female), smoking status (current, not current), body mass index (BMI; kg/m^2^), type 2 diabetes (yes, no), family history of heart disease (yes, no), and education level (less than high school, high school, tertiary education). Covariates were self-reported in the individual's baseline questionnaire.

### 2.4 Statistical analyses

To be included in the analyses, individuals had to have complete data on exposures, covariates, and outcome variables. Baseline differences between included vs excluded, males vs females, MACE vs non-MACE were analysed using χ2 test, Mann Whitney *U* test, and unpaired *t*-test. *P*-values < 0.05 were considered statistically significant.

Before hazard ratios (HRs) and their 95 % *CIs* for the association of sedentary behaviour and physical activity with recurrent cardiovascular events were estimated, the proportionality assumption was checked using the Schoenfeld residuals method. A weak significance was noted for the risk time of the different outcomes. Thus, unadjusted and adjusted (for all covariates) Cox regression models with a time-dependent covariate module were used [22]. Adjusted Cox regressions of physical activity were further adjusted for sedentary behaviour and the sedentary analyses were further adjusted for MVPA. Cox regression models were considered statistically significant if the 95 % CI did not include 1. Differences in HRs between physical activity and sedentary categories were analyzed as interactions based on Altman et al. [23], *CIs* including 0 were interpreted as having no differences. Then the adjusted Cox models were repeated, using quartiles for physical activity and sedentary behaviour data. Sex differences were explored in the fully adjusted Cox regression models by adding an interaction term (sex * exposure) to the analysis and then stratified analyses for males and females. Two sensitivity analyses were performed: 1) Excluding individuals with shorter follow-up time than two years, to reduce the risk of reverse causality; and 2) Excluding individuals self-reporting CHD in wave 1, to assess the risk of a long history of pre-existing heart disease. Adjusted Restricted Cubic Splines were used to explore the linearity of the association between physical activity and sedentary behaviour with recurrent cardiovascular events. Three (location at 0.10, 0.50, and 0.90 percentile), four (location at 0.05, 0.35, 0.65, and 0.95 percentile), and five knots (0.05, 0.275, 0.50, 0.725, and 0.95 percentile) were tested across continuous weekly minutes in physical activity or daily hours in sedentary behaviour. Akaike's Information Criterion was used to identify the best-fit model. Non-linearity (*p* > 0.05) was assessed, with the Wald test. Statistical analyses were performed using SPSS 29.0.1.0 software (IBM, Armonk, NY), and R version 2023.03.1 (R Core Team, 2023) was used for the restricted cubic splines.

## 3 Results

Of the 49,828 individuals with CHD, 40,156 fulfilled the inclusion criteria and were included in analyses (Supplementary 2). Median follow-up time was 8.33 (IQR = 10.04) years. During this period 3260 non-fatal cardiac events, 5161 total cardiac events, and 14,383 MACE were recorded. The mean age was 70 (SD = 10) years and 62 % were men. The majority had a family history of heart disease (62 %) and 16 % were diagnosed with type II diabetes. The mean BMI was 27.2 (SD = 4.7) and 4 % stated they were current smokers. Median time in MVPA was 390 (IQR = 700) min/week, mainly based on walking or moderate-intensity activities. The median daily time in sedentary behaviour was 5 (IQR = 4) h. There were small statistical differences between females and males (Table 1). In general, women had a lower level of education, fewer were diagnosed with type 2 diabetes but more reported a family history of heart disease. There were differences in MVPA and sedentary behaviour with women reporting less time in both, compared to men. There were baseline differences between individuals included vs excluded in the study cohort and between those with vs without a recurrent MACE event, (see Supplementary 3 and 4, respectively). Included individuals, were significantly younger, had higher levels of physical activity, fewer had diabetes, and fewer were current smokers. Meanwhile, individuals having a recurrent MACE were significantly older, more men, smokers, had lower levels of education, diabetes, and had worse levels of physical activity and sedentary behaviour.

**Table 1:** Baseline characteristics of participants.

**Characteristic**	**Total** **(*n*=40,156)**	**Males** **(*n* =24,878)**	**Females** **(*n* =15,278)**
Age *(yr)*, mean (SD)	70.25 (10.25)	70.4 (9.9)	70.0 (10.8)⁎
Tertiary education, number ( %)	8682 (21.6 %)	5901 (23.7 %)	2781 (18.2 %)⁎
Type 2 diabetes, number yes ( %)	6535 (16.3 %)	4348 (17.5 %)	2187 (14.3 %)⁎
BMI *(kg/m^2^)*, mean (SD)	27.24 (4.71)	27.26 (4.18)	27.20 (5.47)
Family history heart disease, number yes ( %)	25,070 (62.4 %)	14,801 (59.5 %)	10,269 (67.2 %)⁎
Current smokers, number yes ( %)	1784 (4.4 %)	1119 (4.5 %)	665 (4.4 %)
Sedentary behavior total (hr/day), median (IQR)	5 (3–7)	5 (3–7)	4 (3–6)⁎
MVPA a (min/wk), median (IQR)	390 (140–840)	390 (150–840)	380 (120–840)⁎
Walking (min/wk), median (IQR)	100 (30–240)	120 (30–240)	90 (20–210)⁎
MPA b (min/wk), median (IQR)	120 (10–403)	120 (10–360)	150 (10–420)⁎
VPA c (min/wk), median (IQR)	0 (0–40)	0 (0–60)	0 (0–20)⁎
Follow-up time (days), median (IQR)	3043 (1354–5022)	3009 (1339–5021)	3098 (1383–5022)
Nonfatal-cardiac events, n ( %)	3260 (8 %)	2301 (9.2 %)	959 (6 %)⁎
Cardiac events including death, n ( %)	5161 (13 %)	3659 (15 %)	1502 (10 %)⁎
Cardiovascular events including death, n ( %)	14,383 (36 %)	9317 (38 %)	5506 (33 %)⁎

a Moderate-to-vigorous intensity physical activity (MVPA).

b Moderate intensity physical activity.

c Vigorous intensity physical activity.

⁎ *p* < 0.05, compared to men.

### 3.1 Association of sedentary behaviour and physical activity with recurrent cardiovascular events

Individuals reporting 7–10.4 hr/day of sedentary behaviour had an approximately 15 % lower risk of recurrent total cardiac events or MACE compared to individuals reporting the highest level (≥ 10.5 h/day) in adjusted analyses (Table 2). The risk was progressively lower with a lower level of sedentary behaviour. There was no significant association between time in sedentary behaviour and the risk of recurrent non-fatal cardiac events.

**Table 2:** Number of events and Hazard ratios (95 % CI) for non-fatal cardiac events, total cardiac events, and major adverse cardiovascular events (MACE) by physical activity and sedentary behavior categories among individuals (*n* = 40,156) with coronary heart disease.

	**Non-fatal cardiac events**					**Total cardiac events event**			**MACE**		
	**n**	**No. of events**		**Unadjusted model**	**Adjusted model**^a^	**No. of events**	Unadjusted model	**Adjusted model**^a^	**No. of events**	**Unadjusted model**	**Adjusted model**^a^
Sedentary behaviour^b^											
≥10.5 hr/day	2272	911		Ref	Ref	1332	Ref	Ref	4047	Ref	Ref
7–10.4 hr/day	7884	1477		0.894 (0.756–1.058)	0.929 (0.785–1.100)	2314	0.790 (0.697–0.896)	0.841 (0.742–0.954)	6325	0.833 (0.770–0.900)	0.856 (0.792–0.926)
3.5–6.9 hr/day	16,683	680	0.871 (0.723–1.050)		0.908 (0.753–1.094)	1158	0.682 (0.592–0.786)	0.738 (0.640–0.851)	3086	0.761 (0.696–0.832)	0.786 (0.718–0.859)
0–3.4 hr/day	13,317	192	0.760 (0.606–0.953)		0.834 (0.665–1.047)	357	0.539 (0.451–0.644)	0.617 (0.517–0.736) e	925	0.693 (0.621–0.774)	0.736 (0.659–0.822) e
Moderate-to-vigorous physical activity^c^											
0 min/wk	2827	267		Ref	Ref	519	Ref	Ref		Ref	Ref
1–149 min/wk	8000	737		0.735 (0.637–0.849)	0.856 (0.744–0.986)	1286	0.648 (0.583–0.720)	0.806 (0.727–0.893)	3199	0.687 (0.642–0.735)	0.790 (0.739–0.844)
150–300 min/wk	6592	571		0.607 (0.515–0.716)	0.820 (0.708–0.951)	864	0.458 (0.404–0.520)	0.693 (0.620–0.774)	2398	0.557 (0.514–0.604)	0.724 (0.676–0.777)
>300 min/wk	22,737	1685		0.469 (0.391–0.563)	0.709 (0.621–0.809)	2492	0.341 (0.296–0.393)	0.595 (0.539–0.656) d	7552	0.468 (0.427–0.512)	0.659 (0.620–0.701) d
Moderate physical activity^c^											
0 min/wk	9318	876		Ref	Ref	1540	Ref	Ref	3815	Ref	Ref
1–149 min/wk	12,173	949		0.710 (0.643–0.78)	0.855 (0.779–0.938)	1453	0.600 (0.555–0.649)	0.787 (0.732–0.846)	4208	0.714 (0.680–0.749)	0.842 (0.806–0.881)
150–300 min/wk	7005	542		0.678 (0.593–0.776)	0.854 (0.766–0.951)	822	0.552 (0.495–0.615)	0.786 (0.721–0.856)	2293	0.647 (0.605–0.692)	0.803 (0.762–0.846)
>300 min/wk	11,660	893		0.627 (0.536–0.734)	0.796 (0.724–0.875)	1346	0.491 (0.432–0.559)	0.709 (0.658–0.764) d	4067	0.640 (0.591–0.692)	0.798 (0.763–0.835)
Walking b											
0 min/wk	7126	687		Ref	Ref	1196	Ref	Ref	2997	Ref	Ref
1–149 min/wk	18,186	1493		0.714 (0.646–0.790)	0.865 (0.789–0.947)	2364	0.641 (0.592–0.693)	0.831 (0.775–0.892)	6553	0.727 (0.692–0.764)	0.850 (0.814–0.888)
150–300 min/wk	8026	687		0.574 (0.496–0.666)	0.758 (0.678–0.848)	860	0.476 (0.422–0.536)	0.696 (0.637–0.760) d	2635	0.618 (0.574–0.665)	0.775 (0.735–0.817) d
>300 min/wk	6818	1493		0.541 (0.447–0.655)	0.750 (0.667–0.843)	741	0.442 (0.377–0.517)	0.689 (0.628–0.756) d	2198	0.576 (0.523–0.634)	0.749 (0.708–0.791) d
Vigorous physical activity c											
0 min/wk	27,232	2373		Ref	Ref	3945	Ref	Ref	10,481	Ref	Ref
1–74 min/wk	5606	413		0.769 (0.684–0.864)	0.927 (0.834–1.031)	567	0.624 (0.565–0.690)	0.843 (0.771–0.921)	1761	0.742 (0.701–0.786)	0.892 (0.847–0.939)
75–150 min/wk	3316	217		0.658 (0.552–0.784)	0.876 (0.761–1.008)	301	0.530 (0.456–0.616)	0.824 (0.732–0.927)	991	0.681 (0.625–0.742)	0.883 (0.827–0.943)
>150 min/wk	4002	257		0.626 (0.509–0.771)	0.818 (0.718–0.932)	348	0.484 (0.403–0.581)	0.753 (0.674–0.841)	1150	0.637 (0.574–0.707)	0.832 (0.782–0.885)
Sedentary behaviour^b^											
≥10.5 hr/day	2272	911		Ref	Ref	1332	Ref	Ref	4047	Ref	Ref
7–10.4 hr/day	7884	1477		0.894 (0.756–1.058)	0.929 (0.785–1.100)	2314	0.790 (0.697–0.896)	0.841 (0.742–0.954)	6325	0.833 (0.770–0.900)	0.856 (0.792–0.926)
3.5–6.9 hr/day	16,683	680	0.871 (0.723–1.050)		0.908 (0.753–1.094)	1158	0.682 (0.592–0.786)	0.738 (0.640–0.851)	3086	0.761 (0.696–0.832)	0.786 (0.718–0.859)
0–3.4 hr/day	13,317	192	0.760 (0.606–0.953)		0.834 (0.665–1.047)	357	0.539 (0.451–0.644)	0.617 (0.517–0.736) e	925	0.693 (0.621–0.774)	0.736 (0.659–0.822) e
Moderate-to-vigorous physical activity (MVPA)/Sedentary behavior (SB)											
MVPA<150 min/wk, SB≥7 hr/day	3228	316	Ref		Ref	658	Ref	Ref	1505	Ref	Ref
MVPA <150 in/wk, SB <7 hr/day	6982	632	0.885 (0.768–1.019)		0.908 (0.788–1.046)	1062	0.701 (0.632–0.777)	0.732 (0.660–0.811)	2698	0.794 (0.742–0.848)	0.807 (0.755–0.863)
MVPA ≥150min/wk,SB ≥7 hr/day	6928	556	0.611 (0.520–0.719)		0.755 (0.641–0.889)	857	0.432 (0.382–0.488)	0.591 (0.523–0.668)	2506	0.569 (0.527–0.615)	0.680 (0.629–0.736)
MVPA ≥150 min/wk, SB <7hr/day	23,018	1756	0.581 (0.485–0.695)		0.708 (0.591–0.848) f	2584	0.383 (0.334–0.439)	0.516 (0.450–0.592) f	7674	0.526 (0.481–0.574)	0.616 (0.564–0.673) f

a All models adjusted for age, sex, education level, body mass index, smoking, type 2 diabetes, family history of heart disease.

b Model also adjusted for Moderate-to-Vigorous Physical Activity.

c Model also adjusted for Sedentary Behaviour.

d lower risk (*p* < 0.05) compared to 1–140 min/week.

e lower risk (*p* < 0.05) compared to 7–10.4 h/day.

f lower risk (*p* < 0.05) compared to MVPA <150 in/week, SB <7 h/day.

Any level of MVPA, moderate-intensity physical activity, or walking showed a lower risk of recurrent non-fatal cardiac events, total cardiac events, and MACE compared to inactivity (0 min/week) in the adjusted model (Table 2). The risk of non-fatal cardiac events, total cardiac events, and MACE were 14 %, 19 %, and 21 % lower respectively, among individuals reporting 1–149 min/week of MVPA compared to inactive. With a progressively lower risk for an even higher category of MVPA. For moderate-intensity physical activity and walking, individuals reporting 1–149 min/week had a 15–21 % lower risk of non-fatal cardiac events, total cardiac events, or MACE compared to inactive, with a progressively lower risk for a higher category described in Table 2. Individuals reporting 1–74 min/week of vigorous-intensity physical activity per week had a 16 % lower risk of total cardiac events and an 11 % lower risk of MACE compared to inactive. For non-fatal cardiac events, individuals reporting ≥ 150 min/week of vigorous-intensity physical activity had an 18 % lower risk compared to inactive individuals. Associations in the fully adjusted models for physical activity and sedentary behaviour were similar when using quartiles instead of the current categories (Supplementary 5).

In the joint associations of time in physical activity and sedentary behaviour, individuals reporting a combination of ≥ 150 min/week in MVPA and < 7 h/day sedentary behaviour had a 29 % lower risk of non-fatal cardiac events, 48 % lower risk of total cardiac events and 38 % lower risk of MACE compared to the reference group (<150 min/week of MVPA and ≥7 h/day of sedentary behaviour). For total cardiac events and MACE, all categories had a lower risk compared to the reference group. Meanwhile, for non-fatal cardiac events, the individuals had to reach ≥ 150 min/week of MVPA to have a significantly lower risk.

The independent and joint association of sedentary behaviour and physical activity with the outcomes was similar for men and women (Supplementary 6). However, the protective effect of walking and vigorous-intensity physical activity on the risk of non-fatal cardiac events and total cardiac events is more substantial in women than men, with a more pronounced lower risk than observed in men. There were no sustainable differences in the association between sedentary behaviour and physical activity with risk non-fatal -, total cardiac events, and MACE among individuals with at least two years of follow-up compared with the total cohort (Supplementary 7). When excluding individuals from the first wave, the HRs were similar to the total analytic sample, although all significant differences did not persist due to wide confidence intervals (Supplementary 8).

### 3.2 Shape of the curve of the association of sedentary behaviour and physical activity with cardiovascular events

**Fig. 1:**
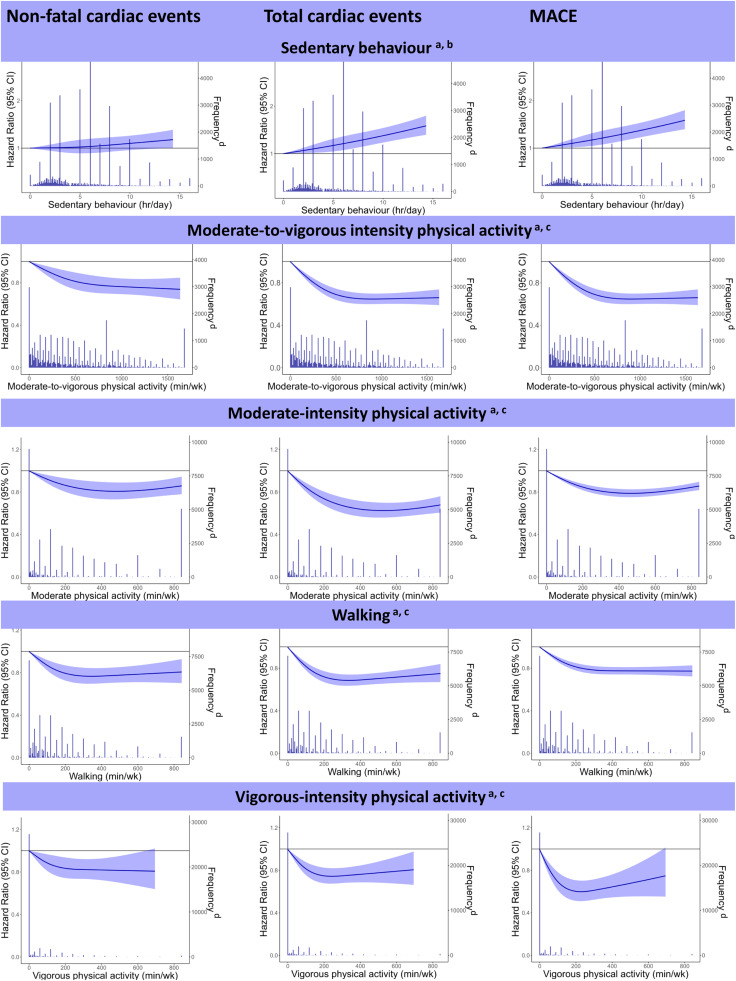
Associations of time in sedentary behaviour, walking, moderate-, vigorous- and moderate-to-vigorous intensity physical activity with the risk of non-fatal cardiac events, total cardiac events, and major adverse cardiovascular events (MACE) in individuals with coronary heart disease (*n* = 40,156). ^a^ All models adjusted for age, sex, education level, body mass index, smoking, type 2 diabetes, family history of heart disease, ^b^ Model also adjusted for Moderate-to-Vigorous Physical Activity, ^c^ Model also adjusted for Sedentary Behaviour, ^d^ Frequency is the number of individuals.

Hours in sedentary behaviour were linearly associated with the risk of non-fatal cardiac events (nonlinearity, *p* = 0.587) and total cardiac events (nonlinearity, *p* = 0.953), indicating no maximal effect of decreasing sedentary behaviour (Fig. 1). Meanwhile, the association with MACE was curvilinear (nonlinearity, *p* = 0.017). The associations between minutes in MVPA, moderate-intensity physical activity, and walking with the risk of non-fatal cardiac events, total cardiac events, and MACE, were curvilinear (nonlinearity, *p* < 0.05). This indicates that there was no additional benefit above a certain level of physical activity. For minutes in vigorous-intensity physical activity, the association was linear with the risk of non-fatal cardiac events (nonlinearity, *p* = 0.065), but curvilinear for total cardiac events and MACE (nonlinearity, *p* < 0.001). Results are based on restricted cubic splines with three knots, increasing the number of knots did not impact the linearity.

## 4 Discussion

The main findings from this large cohort of individuals with CHD are that a higher level of physical activity is associated with a lower risk of recurrent cardiovascular events. Further, a lower level of sedentary behaviour is associated with a lower risk of total cardiac events and MACE but not with non-fatal cardiac events. Importantly, the lowest risk was among individuals who were able to achieve, ≥ 150 min/week of MVPA and < 7 h/day sedentary behaviour, compared to the most inactive individuals.

No previous studies have, to our knowledge, explored the association between time in sedentary behaviour and risk of recurrent cardiovascular events among patients with CHD. Interestingly, our study indicate that a lower level of sedentary behaviour is of greater importance to decrease the risk of more severe cardiovascular events. Finding a 16–21 % lower risk for total cardiac events and MACE for individuals reporting 3.5–6.9 h/day compared to individuals reporting ≥ 10.5 h/day, but no significantly lower risk of a non-fatal cardiac event. Two smaller studies among individuals with CHD have explored the association between sedentary behaviour with all-cause mortality and total hospital readmissions. Finding a 62 % lower risk of all-cause mortality among individuals reporting < 4 h/day of sedentary behaviour compared to 4–7 h/day [24] and a 42 % lower risk of total hospital readmission for <7 h/day compared to ≥ 10 h/day [9], which is more prominent compared to our results. Together, this indicates that limiting time in sedentary behaviour may be of greater importance to decrease the risk of severe cardiovascular events or non-diagnose specific outcomes compared to non-fatal cardiac events.

Individuals reporting 1–149 min/week of MVPA were associated with a 14–21 % lower risk of recurrent cardiovascular events compared to inactive individuals. The lower risk was more prominent for total cardiac events, and MACE, compared to non-fatal cardiac events. The pattern was homogenous for walking and activities at moderate- and vigorous-intensity with the risk of a recurrent cardiovascular event. Similar associations were seen in the large STABILITY study among individuals with CHD from 39 countries. They found a significantly lower risk of MACE and mortality among individuals with higher levels of physical activity, but no lower risk of recurrent MI or stroke [8].

Importantly, the joint association of MVPA and limited time in sedentary behaviour had the lowest risk of all recurrent cardiovascular events. However, if the physical activity guidelines are met, a lower level of sedentary behaviour did not significantly affect the risk of a nonfatal cardiac event. Thus, regular MVPA seems to be of greater importance and partly modifies the risk of ≥7 h/day of sedentary behaviour with the risk of a recurrent cardiovascular event. The observation that a higher level of physical activity modifies the risk of time in sedentary behaviour has previously been seen for CVD morbidity and mortality in the general population [25,26]. The stronger association with regular physical activity may be due to its positive effect on several CVD risk factors e.g., blood pressure, lipid level, the vascular system, and metabolic health [21]. However, individuals who cannot reach ≥ 150 min/week in MVPA but are limiting their time in sedentary behaviour, still have a lower risk of both total cardiac events and MACE, compared to individuals with ≥7 h/day in sedentary behaviour. This emphasizes the importance of supporting individuals to decrease sedentary time if they cannot reach the public health guidelines of ≥ 150 min of physical activity, which represents a large proportion of individuals with heart disease [27].

We found a linear association between time in sedentary behaviour and non-fatal and total cardiac events, with no maximal effect of lower time. There are to our knowledge no other studies that have explored the shape of the association among individuals with CHD, but our results are supported by a large meta-analysis in the general population, finding a linear association between time in sedentary behaviour and all-cause mortality [20]. This is in contrast to MVPA, with our study finding a curvilinear association with cardiovascular events, with the greatest decline in risk for the first minutes of physical activity. However, exceeding the guidelines of ≥150 min/week leads to a further reduced risk for all cardiovascular events in individuals with CHD. The curvilinear association was also seen for walking and activities at moderate-, and vigorous-intensity. A study from The Netherlands among individuals with cardiovascular disease supports our results with a curvilinear association between leisure-time physical activity and risk of recurrent cardiovascular events [10]. However, Bakker et al. found a linear association between MVPA and risk of MACE among individuals with cardiovascular diseases [13]. Therefore, further exploration of this association is indicated due to these contrasting results in individuals with cardiovascular disease including CHD.

### 4.1 Strengths and limitations

A major strength of this prospective cohort study is the size of the study cohort, permitting adjustment for numerous potential confounders and allowing for stratified analyses. To reduce the risk of reversed causality, sensitvity analysis excluding patients with shorter follow-up times than 2 years were performed with only minor differences for risk of cardiovascular events compared with the total cohort. This may suggest that the effect of reverse causation was limited. Included individuals had to have complete data of covariates, and exposure and outcome variables, increasing internal validity. There were statistical differences in baseline characteristics between included and excluded individuals, with the excluded being older, women, current smokers, had diabetes, and lower levels of physical activity to a greater extent. This contributes to a potentially lower external validity for this population. A limitation is that it is based on individuals self-reporting heart disease. Although, previous studies have shown that a self-reported history of cardiovascular disease is a valid measure of diagnosed disease [28,29]. Thereto, physical activity and sedentary behaviour were self-reported, with an increased risk of recall and social desirability bias [30]. However, the Active Australia Survey is valid to assess time in MVPA compared to accelerometers [19]. Another potential limitation is that the level of physical activity and sedentary behaviour may fluctuate over time, and the reported levels in the surveys may not represent their physical activity and sedentary behaviour over time.

## 5 Conclusion

A higher level of physical activity and a lower level of sedentary behaviour appears to lower the risk of cardiovascular events in individuals with CHD. The lowest risk of total cardiac events and MACE were found for the joint association of achieving the physical activity guidelines and limiting sedentary behaviour to < 7 h/day. However, MVPA seems to be of greater importance in lowering the risk, and partly modifies the risk of sedentary behaviour in the joint association. Together, this emphasizes the importance of healthcare professionals providing recommendations of primarily physical activity. Limiting time in sedentary behaviour has additional effects on individuals with CHD to lower their risk of recurrent cardiovascular events, especially among individuals with the lowest level of physical activity (Central illustration).

**Central illustration:**
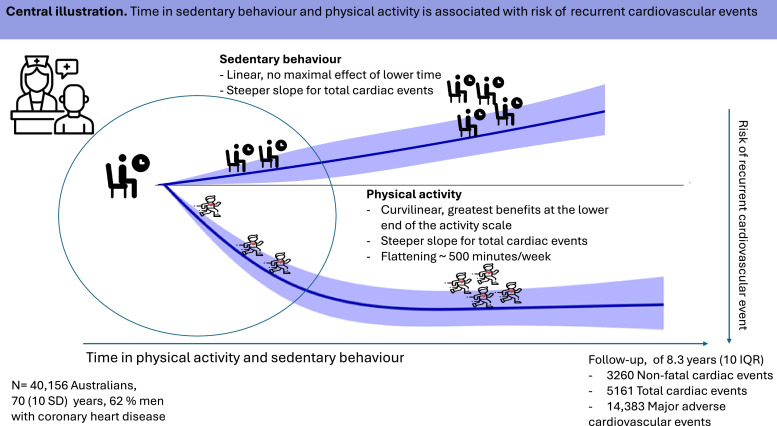
Time in sedentary behaviour and physical activity is associated with risk if recurrent cardiovascular events.

## Data sharing statement

The datasets used and analysed during the study are not available as they are third party data not owned or collected by the authors. However, the data are available from the data custodians for approved research projects. Data access enquiries can be made to the Sax Institute https://www.saxinstitute.org.au/solutions/45-and-up-study/use-the-45-and-up-study/apply-for-access/.

## Author agreement

All authors should have made substantial contributions to all of the following:

1. The conception and design of the study, or acquisition of data, or analysis and interpretation of data.
2. Drafting the article or revising it critically for important intellectual content.
3. Final approval of the version to be submitted.

All authors should agree to be accountable for all aspects of the work to ensure that the questions related to the accuracy or integrity of any part of the work are appropriately investigated and resolved.

## Funding

Funding for this study was provided by a University of Canberra Faculty of Health Seed Grant and the University of Canberra Health Research Institute. NF is partly funded by the 2021 Medical Research Future Fund Cardiovascular Health Mission Grant Scheme (ID 2015,953). AL is supported by a Swedish Heart-Lung Foundation Post-doctoral Research Fellow (Abroad) Scholarship (nr. 20,220,860).

## CRediT authorship contribution statement

**Amanda Lönn:** Conceptualization, Data curation, Formal analysis, Funding acquisition, Methodology, Visualization, Writing – original draft. **Suzanne J Carroll:** Conceptualization, Data curation, Methodology, Writing – review & editing, Investigation. **Theo Niyonsenga:** Conceptualization, Formal analysis, Methodology, Writing – review & editing, Investigation. **Adrian Bauman:** Conceptualization, Investigation, Supervision, Writing – review & editing. **Rachel Davey:** Conceptualization, Funding acquisition, Investigation, Resources, Writing – review & editing. **Robyn Gallagher:** Conceptualization, Investigation, Writing – review & editing. **Nicole Freene:** Conceptualization, Funding acquisition, Investigation, Methodology, Project administration, Supervision, Writing – review & editing, Resources.

## Declaration of competing interest

The authors declare that they have no known competing financial interests or personal relationships that could have appeared to influence the work reported in this paper.

## Appendix Supplementary materials

Image, application 1

Image, application 2

Image, application 3

Image, application 4

Image, application 5

Image, application 6

Image, application 7

Image, application 8
