# Supplementary material for: Independent and joint associations of sedentary behaviour and physical activity with risk of recurrent cardiovascular events in 40,156 Australian adults with coronary heart disease
Source: Am J Prev Cardiol. 2025 Apr 17;22:100998. doi: 10.1016/j.ajpc.2025.100998 (PMC12041785; doi:10.1016/j.ajpc.2025.100998)
Supplement: Supplementary file 2 [file mmc2.docx]

Supplementary 1. Physcial Activity and Sedentary Behaviour questions asked in the 45 and Up Study.

| Questions | Wave 1 | SEEF | Wave 2 | Wave 3 |
| --- | --- | --- | --- | --- |
| Physical Activity |  |  |  |  |
| How many TIMES did you do each of these activities LAST WEEK?  *(put "0" if you did not do this activity)*  Walking continuously, for at least 10 minutes  *(for recreation or exercise or to get to or from places)*  Vigorous physical activity  *(that made you breathe harder or puff and pant, like jogging,cycling, aerobics, competitive tennis, but not household chores or gardening)*  Moderate physical activity  *(like gentle swimming, social tennis, vigorous gardening or work around the house)* | x | x | x | x |
| If you add up all the time you spent doing each activity LAST WEEK, how much time did you spend ALTOGETHER doing each type of activity (mins or hrs)?  *(put “0” if you did not do this activity)*  Walking continuously, for at least 10 minutes  *(for recreation or exercise or to get to or from places)*  Vigorous physical activity  *(that made you breathe harder or puff and pant, like jogging, cycling, aerobics, competitive tennis, but not household chores or gardening)*  Moderate physical activity  *(like gentle swimming, social tennis, vigorous gardening or work around the house)* | x | x | x | x |
| Sedentary Behaviour |  |  |  |  |
| About how many HOURS in each 24 hour DAY do you usually spend doing the following?  *(please put “0” if you do not spend any time doing it)*  Sitting  Watching television or using a computer | x | x |  |  |
| During the LAST 7 DAYS, how much time did you spend SITTING on a usual WEEK day and a usual WEEKEND day (mins or hrs):  (write your answers in the spaces provided)  for TRANSPORT (e.g. in car, bus, train etc)  at WORK (e.g. sitting at desk or using a computer)  watching TV  using a computer at home (e.g. email, games, information, chatting)  other leisure activities (e.g. socialising, movies etc but NOT including TV or computer use) |  |  | x | x |
