# Supplementary material for: Independent and joint associations of sedentary behaviour and physical activity with risk of recurrent cardiovascular events in 40,156 Australian adults with coronary heart disease
Source: Am J Prev Cardiol. 2025 Apr 17;22:100998. doi: 10.1016/j.ajpc.2025.100998 (PMC12041785; doi:10.1016/j.ajpc.2025.100998)
Supplement: Supplementary file 3 [file mmc3.docx]

**Supplementary 3.** Baseline characteristics of individuals included and excluded in the study cohort

| **Characteristic** | **Excluded**  **(n = 9625)** | **Included**  **(n = 40,156)** |
| --- | --- | --- |
| Age *(yr)*, mean (SD) | 73.03 (10.41) | 70.25 (10.25)* |
| Sex, men | 5168 (53.7) | 24878 (62%)* |
| Tertiary education, number (%) | 1298 (13.5%) | 8682 (21.6%)* |
| Type 2 diabetes, number yes (%) | 2038 (21.3%) | 6535 (16.3%)* |
| BMI *(kg/m^2^)*, mean (SD) | 27.14 (4.92) | 27.24 (4.71) |
| Family history heart disease, number yes (%) | 5753 (59.9%) | 25,070 (62.4%)* |
| Current smokers, number yes (%) | 465 (5%) | 1784 (4.4%)* |
| Sedentary behavior total (hr/day), median (q1-q3) | 5 (3-7) | 5 (3-7) |
| MVPA (min/wk), median (q1-q3) | 270 (60-720) | 390 (140-840) * |
| Walking (min/wk), median (q1-q3) | 60 (0-210) | 100 (30-240) * |
| MPA (min/wk), median (q1-q3) | 70 (0-300) | 120 (10-403) * |
| VPA (min/wk), median (q1-q3) | 0 (0-00) | 0 (0-40) * |

^a^ Moderate-to-vigorous intensity physical activity (MVPA)

^b^ Moderate intensity physical activity

^c^ Vigorous intensity physical activity

* p < 0.05, compared to individuals with recurrent excluded
