# Supplementary material for: Independent and joint associations of sedentary behaviour and physical activity with risk of recurrent cardiovascular events in 40,156 Australian adults with coronary heart disease
Source: Am J Prev Cardiol. 2025 Apr 17;22:100998. doi: 10.1016/j.ajpc.2025.100998 (PMC12041785; doi:10.1016/j.ajpc.2025.100998)
Supplement: Supplementary file 4 [file mmc4.docx]

**Supplementary 4**. Baseline characteristics of individuals with and without a recurrent major adverse cardiac event (MACE) during follow-up

| **Characteristic** | **Recurrent MACE**  **(n =14.383 )** | **No recurrent MACE (n= 33,773)** |
| --- | --- | --- |
| Age *(yr)*, mean (SD) | 72 (11) | 69 (10)* |
| Sex, men, number (%) | 9317 (65) | 15,561 (60) * |
| Tertiary education, number (%) | 2356 (18) | 6146 (24) * |
| Type 2 diabetes, number yes (%) | 2670 (19) | 3865 (15) * |
| BMI *(kg/m^2^)*, mean (SD) | 27.1 (5) | 27.3 (5) * |
| Family history heart disease, number yes (%) | 8936 (62) | 16 134 (63) |
| Current smokers, number yes (%) | 707 (5) | 1077 (4) * |
| Sedentary behavior total (hr/day), median (q1-q3) | 5 (3-7) | 4.0 (2.9-6.3) * |
| MVPA (min/wk), median (q1-q3) | 355 (120-780) | 420 (160-840) * |
| Walking (min/wk), median (q1-q3) | 90 (20-210) | 120 (30-240) * |
| MPA (min/wk), median (q1-q3) | 120 (0-360) | 120 (20-420) * |
| VPA (min/wk), median (q1-q3) | 0 (0-15) | 0 (0-60) * |

^a^ Moderate-to-vigorous intensity physical activity (MVPA)

^b^ Moderate intensity physical activity

^c^ Vigorous intensity physical activity

* p < 0.05, compared to individuals with recurrent MACE
