# Supplementary material for: Independent and joint associations of sedentary behaviour and physical activity with risk of recurrent cardiovascular events in 40,156 Australian adults with coronary heart disease
Source: Am J Prev Cardiol. 2025 Apr 17;22:100998. doi: 10.1016/j.ajpc.2025.100998 (PMC12041785; doi:10.1016/j.ajpc.2025.100998)
Supplement: Supplementary file 5 [file mmc5.docx]

**Supplementary 5**. Number of events and Hazard ratios (95% CI) for non-fatal cardiac events, total ischemic cardiac events, and major adverse cardiovascular event (MACE) by physical activity and sedentary behavior quartiles individuals (n=40,156) with coronary heart disease

|  | **Non-fatal cardiac events** | | | | **Total cardiac events** | | | **MACE** | | |
| --- | --- | --- | --- | --- | --- | --- | --- | --- | --- | --- |
|  | **n** | **No. of events** | **Unadjusted model** | **Adjusted model^a^** | **No. of events** | **Unadjusted model** | **Adjusted model^a^** | **No. of events** | **Unadjusted model** | **Adjusted model^a^** |
| **Sedentary behaviour^b^** | | |  |  |  |  |  |  |  |  |
| ≥ 7 hours/day | 10156 | 872 | **Ref** | **Ref** | 1515 | **Ref** | **Ref** | 4011 | Ref | Ref |
| 5-6.99 hours/day | 9977 | 888 | 0.967 (0.871-1.072) | 0.963 (0.868-1.069) | 1412 | 0.867 (0.799-0.941) | 0.872 (0.803-0.946) | 3817 | 0.917 (0.872-0.964) | 0.909 (0.864-0.956) |
| 3-4.99 hours/day | 10777 | 924 | 0.876 (0.768-0.999) | 0.895 (0.784-1.021) | 1403 | 0.735 (0.661-0.817) | 0.762 (0.685-0.848) | 4001 | 0.851 (0.797-0.908) | 0.859 (0.805-0.917) |
| ≤ 2.99 hours/day | 9238 | 576 | 0.774 (0.653-0.917) | 0.818 (0.691-0.970) | 831 | 0.611 (0.532-0.703) | 0.651 (0.566-0.748) | 2554 | 0.795 (0.731-.865) | 0.810 (0.744-0.881) |
| **Moderate-to-vigorous physical activity^c^** | | |  |  |  |  |  |  |  |  |
| ≤ 139.9 min/wk | 9915 | 930 | **Ref** | **Ref** | 1678 | **Ref** | **Ref** | 4099 | Ref | Ref |
| 140-389.9 min/wk | 9941 | 820 | 0.733 (0.663-0.812) | 0.864 (0.786-0.950) | 1270 | 0.616 (0.569-0.667) | 0.779 (0.724-0.839) | 3543 | 0.718 (0.683-0.754) | 0.828 (0.791-0.866) |
| 390-839.9 min/wk | 10139 | 755 | 0.614 (0.541-0.697) | 0.795 (0.721-0.877) | 1096 | 0.474 (0.427-0.525) | 0.683 (0.633-0.738) | 3370 | 0.625 (0.587-0.666) | 0.778 (0.743-0.815) |
| ≥ 840 min/wk | 10161 | 755 | 0.582 (0.495-0.684) | 0.780 (0.707-.860) | 1117 | 0.448 (0.393-0.511) | 0.681 (0.630-0.735) | 3371 | 0.598 (0.552-0.649) | 0.766 (0.732-0.802) |
| **Moderate physical activity^c^** | |  |  |  |  |  |  |  |  |  |
| ≤ 9.99 min/wk | 10243 | 956 | **Ref** | **Ref** | 1676 | **Ref** | **Ref** | 4183 | Ref | Ref |
| 10-119.9 min/wk | 10523 | 823 | 0.716 (0.647-0.792) | 0.877 (0.798-0.964) | 1233 | 0.593 (0.547-0.642) | 0.795 (0.738-0.856) | 3582 | 0.704 (0.671-0.740) | 0.840 (0.803-0.879) |
| 120-402.9 min/wk | 9351 | 691 | 0.647 (0.569-0.735) | 0.819 (0.742-0.904) | 1074 | 0.538 (0.486-0.596) | 0.767 (0.710-0.828) | 3075 | 0.648 (0.608-0.691) | 0.799 (0.762-0.837) |
| ≥ 403 min/wk | 10039 | 790 | 0.647 (0.552-0.759) | 0.825 (0.750-0.908) | 1178 | 0.499 (0.438-0.568) | 0.724 (0.671-0.780) | 3543 | 0.650 (0.600-0.704) | 0.806 (0.770-0.843) |
| **Walking^b^** |  |  |  |  |  |  |  |  |  |  |
| ≤ 29.9 min/wk | 9662 | 925 | **Ref** | **Ref** | 1613 | **Ref** | **Ref** | **4005** | Ref | Ref |
| 30-99.9 min/wk | 10041 | 818 | 0.740 (0.668-0.820) | 0.866 (0.788-0.952) | 1287 | 0.658 (0.607-0.713) | 0.818 (0.760-0.881) | 3621 | 0.759 (0.723-0.798) | 0.865 (0.827-0.905) |
| 100-239.9 min/wk | 9717 | 749 | 0.657 (0.578-0.746) | 0.830 (0.753-0.915) | 1122 | 0.549 (0.495-0.608) | 0.757 (0.701-0.818) | 3310 | 0.679 (0.637-0.723) | 0.823 (0.785-0.862) |
| ≥ 240 min/wk | 10736 | 768 | 0.577 (0.492-0.678) | 0.742 (0.674-0.818) | 1139 | 0.471 (0.413-0.537) | 0.673 (0.623-0.727) | 3447 | 0.612 (0.565-0.664) | 0.759 (0.725-0.795) |
| **Vigorous physical activity^c^** | |  |  |  |  |  |  |  |  |  |
| ≤ 39.9 min/wk (Q 1-3) | 30381 | 2617 | **Ref** | **Ref** | 4285 | **Ref** | **Ref** | 11500 | Ref | Ref |
| ≥ 40 min/wk (Q 4) | 9775 | 643 | 0.661 (0.568-0.770) | 0.860 (0.788-0.940) | 876 | 0.540 (0.473-0.617) | 0.798 (0.741-0.860) | 2883 | 0.696 (0.645-0.751) | 0.870 (0.834-0.907) |
| **Moderate-to-vigorous physical activity (MVPA) /Sedentary behavior (SB)** | | | | |  |  |  |  |  |  |
| Quartile 1 MVPA (least) |  |  |  |  |  |  |  |  |  |  |
| ≥ 7 hours/day | 3143 | 312 | **Ref** | **Ref** | 644 | **Ref** | **Ref** | 1474 | Ref | Ref |
| 5-6.99 hours/day | 2323 | 253 | 1.012 (0.842-1.215) | 1.013 (0.843-1.216) | 435 | 0.847 (0.739-0.970) | 0.859 (0.750-0.985) | 1024 | 0.852 (0.779-0.931) | 0.852 (0.779-0.931) |
| 3-4.99 hours/day | 2282 | 214 | 0.801 (0.632-1.016) | 0.819 (0.646-1.039) | 361 | 0.661 (0.552-0.793) | 0.692 (0.576-0.830) | 933 | 0.714 (0.635-0.802) | 0.730 (0.649-0.821) |
| ≤ 2.99 hours/day | 2164 | 151 | 0.715 (0.530-0.964) | 0.729 (0.541-0.982) | 238 | 0.557 (0.442-0.703) | 0.567 (0.450-0.714) | 668 | 0.652 (0.562-0.757) | 0.654 (0.563-0.759) |
| Quartile 2 MVPA |  |  |  |  |  |  |  |  |  |  |
| ≥ 7 hours/day | 2695 | 221 | **Ref** | **Ref** | 362 | **Ref** | **Ref** | 1007 | Ref | Ref |
| 5-6.99 hours/day | 2454 | 216 | 1.065 (0.862-1.315) | 0.992 (0.803-1.225) | 349 | 1.030 (0.871-1.218) | 0.937 (0.792-1.108) | 938 | 1.053 (0.950-1.167) | 0.992 (0.895-1.099) |
| 3-4.99 hours/day | 2637 | 244 | 1.102 (0.845-1.436) | 1.031 (0.790-1.344) | 360 | 0.954 (0.767-1.187) | 0.872 (0.700-1.085) | 980 | 1.046 (0.915-1.197) | 0.984 (0.860-1.125) |
| ≤ 2.99 hours/day | 2153 | 139 | 0.946 (0.671-1.336) | 0.943 (0.668-1.331) | 199 | 0.792 (0.594-1.056) | 0.766 (0.575-1.021) | 618 | 1.046 (0.879-1.244) | 1.004 (0.844-1.194) |
| Quartile 3 MVPA |  |  |  |  |  |  |  |  |  |  |
| ≥ 7 hours/day | 2406 | 176 | **Ref** | **Ref** | 260 | **Ref** | **Ref** | 853 | **Ref** | **Ref** |
| 5-6.99 hours/day | 2592 | 210 | 1.040 (0.831-1.302) | 0.986 (0.787-1.235) | 316 | 1.026 (0.852-1.235) | 0.945 (0.785-1.138) | 935 | 1.002 (0.900-1.115) | 0.947 (0.851-1.054) |
| 3-4.99 hours/day | 2806 | 225 | 0.975 (0.732-1.299) | 0.938 (0.705-1.250) | 327 | 0.898 (0.705-1.144) | 0.829 (0.651-1.056) | 981 | 0.966 (0.840-1.112) | 0.911 (0.791-1.049) |
| ≤ 2.99 hours/day | 2334 | 144 | 0.938 (0.648-1.357) | 0.944 (0.652-1.366) | 193 | 0.780 (0.566-1.073) | 0.754 (0.548-1.038) | 601 | 0.934 (0.778-1.121) | 0.892 (0.743-1.070) |
| Quartile 4 MVPA (most) |  |  |  |  |  |  |  |  |  |  |
| ≥ 7 hours/day | 1912 | 163 | **Ref** | **Ref** | 249 | **Ref** | **Ref** | 677 | **Ref** | **Ref** |
| 5-6.99 hours/day | 2608 | 209 | 0.895 (0.713-1.125) | 0.879 (0.699-1.104) | 312 | 0.862 (0.714-1.040) | 0.841 (0.697-1.015) | 920 | 0.954 (0.853-1.067) | 0.927 (0.828-1.037) |
| 3-4.99 hours/day | 3052 | 241 | 0.845 (0.635-1.125) | 0.850 (0.638-1.133) | 355 | 0.791 (0.622-1.007) | 0.799 (0.627-1.018) | 1107 | 0.947 (0.820-1.093) | 0.940 (0.814-1.086) |
| ≤ 2.99 hours/day | 2587 | 142 | 0.721 (0.498-1.044) | 0.761 (0.525-1.102) | 201 | 0.650 (0.473-0.893) | 0.693 (0.505-0.951) | 667 | 0.850 (0.705-1.024) | 0.863 (0.716-1.040) |

^a^ All models adjusted for age, sex, education level, body mass index, smoking, type 2 diabetes, family history of heart disease

^b^ Model also adjusted for Sedentary Behaviour

^c^ Model also adjusted for Moderate-to-Vigorous Physical Activity
