# Supplementary material for: Independent and joint associations of sedentary behaviour and physical activity with risk of recurrent cardiovascular events in 40,156 Australian adults with coronary heart disease
Source: Am J Prev Cardiol. 2025 Apr 17;22:100998. doi: 10.1016/j.ajpc.2025.100998 (PMC12041785; doi:10.1016/j.ajpc.2025.100998)
Supplement: Supplementary file 6 [file mmc6.docx]

**Supplementary 6.** Number of events and Hazard ratios (95% CI) for non-fatal cardiac events, total cardiac events, and major adverse cardiovascular event (MACE) by physical activity and sedentary behavior among individuals with ≥ 2 years of follow-up (n= 34,520)

|  | **Non-fatal cardiac event^a^** | | | **Total cardiac events** | | **MACE** | |
| --- | --- | --- | --- | --- | --- | --- | --- |
|  | **N= 34.520** | **No. of events** | **HRs (95% CI)** | **No. of events** | **HRs (95% CI)** | **No. of events** | **HRs (95% CI)** |
| **Sedentary behavior^b^** | | | |  |  |  |  |
| ≥10.5 hr/day | 1912 | 146 | Ref | 268 | Ref | 268 | Ref |
| 7-10.4 hr/day | 6937 | 508 | 0.869 (0.707-1.068) | 907 | 0.805 (0.691-0.938) | 907 | 0.805 (0.691-0.938) |
| 3.5-6.9 hr/day | 14747 | 1135 | 0.863 (0.667-1.117) | 1837 | 0.683 (0.562-0.830) | 1837 | 0.683 (0.562-0.830) |
| 0-3.4 hr/day | 10926 | 681 | 0.812 (0.580-1.136) | 1024 | 0.561 (0.433-0.728) | 1024 | 0.561 (0.433-0.728) |
| **Moderate-to-vigorous physical activity^c^** |  |  |  |  |  |  |  |
| 0 min/wk | 2201 | 174 | Ref | 358 | Ref | 358 | Ref |
| 1-149 min/wk | 6828 | 547 | 0.902 (0.760-1.071) | 997 | 0.840 (0.744-0.949) | 997 | 0.840 (0.744-0.949) |
| 150-300 min/wk | 5693 | 430 | 0.858 (0.718-1.026) | 673 | 0.712 (0.625-0.811) | 673 | 0.712 (0.625-0.811) |
| >300 min/wk | 19800 | 1319 | 0.762 (0.648-0.895) | 2008 | 0.623 (0.555-0.699) | 2008 | 0.623 (0.555-0.699) |
| **Moderate physical activity^c^** | | | |  |  |  |  |
| 0 min/wk | 7747 | 629 | Ref | 1153 | Ref | 1153 | Ref |
| 1-149 min/wk | 10583 | 739 | 0.889 (0.798-0.990) | 1151 | 0.797 (0.734-0.866) | 1151 | 0.797 (0.734-0.866) |
| 150-300 min/wk | 6016 | 419 | 0.880 (0.777- 0.997) | 653 | 0.797 (0.723-0.878) | 653 | 0.797 (0.723-0.878) |
| >300 min/wk | 10176 | 683 | 0.804 (0.721-0.897) | 1079 | 0.716 (0.658-0.779) | 1079 | 0.716 (0.658-0.779) |
| **Walking^c^** |  |  |  |  |  |  |  |
| 0 min/wk | 5832 | 475 | Ref | 870 | Ref | 870 | Ref |
| 1-149 min/wk | 5924 | 1134 | 0.916 (0.822-1.021) | 1854 | 0.866 (0.799-0.940) | 1854 | 0.866 (0.799-0.940) |
| 150-300 min/wk | 15718 | 464 | 0.838 (0.736-0.953) | 701 | 0.745 (0.674-0.824) | 701 | 0.745 (0.674-0.824) |
| >300 min/wk | 6951 | 397 | 0.817 (0.714-0.935) | 611 | 0.742 (0.668-0.824) | 611 | 0.742 (0.668-0.824) |
| **Vigorous physical activity ^c^** | | | |  |  |  |  |
| 0 min/wk | 23326 | 1776 | Ref | 3063 | Ref | 3063 | Ref |
| 1-74 min/wk | 4844 | 329 | 0.964 (0.855-1.086) | 461 | 0.867 (0.785-0.958) | 461 | 0.867 (0.785-0.958) |
| 75-150 min/wk | 2877 | 166 | 0.870 (0.741-1.022) | 234 | 0.808 (0.707-0.924) | 234 | 0.808 (0.707-0.924) |
| >150 min/wk | 3475 | 199 | 0.820 (0.707-0.951) | 278 | 0.757 (0.669-0.857) | 278 | 0.757 (0.669-0.857) |
| **Moderate-to-vigorous physical activity (MVPA)/Sedentary behavior (SB)** | | | | |  |  |  |
| MVPA <150 min/wk, SB ≥7 hr/day | 2682 | 225 | Ref | 496 | Ref | 496 | Ref |
| MVPA <150 min/wk, SB <7 hr/day | 5795 | 451 | 1.094 (0.620-1.929) | 788 | 0.701 (0.618-0.795) | 788 | 0.701 (0.618-0.795) |
| MVPA ≥150 min/wk, SB ≥7 hr/day | 6167 | 429 | 0.735 (0.374-1.445) | 679 | 0.557 (0.474-0.655) | 679 | 0.557 (0.474-0.655) |
| MVPA ≥150 min/wk, SB <7 hr/day | 19878 | 1365 | 0.943 (0.482-1.847) | 2073 | 0.486 (0.398-0.595) | 2073 | 0.486 (0.398-0.595) |

^a^ All models adjusted for age, education level, BMI, smoking, type 2 diabetes, family history of heart disease, ^b^ Model also adjusted for Sedentary Behaviour, ^c^ Model also adjusted for MVPA
