# Supplementary material for: Independent and joint associations of sedentary behaviour and physical activity with risk of recurrent cardiovascular events in 40,156 Australian adults with coronary heart disease
Source: Am J Prev Cardiol. 2025 Apr 17;22:100998. doi: 10.1016/j.ajpc.2025.100998 (PMC12041785; doi:10.1016/j.ajpc.2025.100998)
Supplement: Supplementary file 7 [file mmc7.docx]

**Supplementary 7**. Number of events and Hazard ratios (95% CI) for non-fatal cardiac events, total cardiac events, and major adverse cardiovascular event (MACE) by physical activity and sedentary behavior categories among individuals with coronary heart disease excluding participants from wave 1

|  | **Non-fatal cardiac events^a^** | | | **Total cardiac events^a^** | | **MACE** | |
| --- | --- | --- | --- | --- | --- | --- | --- |
|  | **N=11,006** | **No. of events** | **HRs (95% CI)** | **No. of events** | **HRs (95% CI)** | **No. of events** | **HRs (95% CI)** |
| **Sedentary behavior^b^** | | | |  |  |  |  |
| ≥10.5 hr/day | 411 | 8 | Ref | 12 | Ref | 64 | Ref |
| 7-10.4 hr/day | 1148 | 33 | 1.326 (0.605-2.909) | 36 | 0.977 (0.501-1.905) | 180 | 1.019 (0.758-1.370) |
| 3.5-6.9 hr/day | 3129 | 107 | 1.422(0.643-3.142) | 128 | 1.160 (0.592-2.272) | 568 | 1.288 (0.945-1.756) |
| 0-3.4 hr/day | 6318 | 204 | 1.326 (0.551-3.190) | 259 | 1.114 (0.523-2.372) | 1090 | 1.406 (0.988-2.003) |
| **Moderate-to-vigorous physical activity^c^** | |  |  |  |  |  |  |
| 0 min/wk | 829 | 40 | Ref | 57 | Ref | 188 | Ref |
| 1-149 min/wk | 1820 | 55 | 0.626 (0.416-0.943) | 78 | 0.640 (0.454-0.902) | 334 | 0.766 (0.641-0.917) |
| 150-300 min/wk | 1671 | 66 | 0.856 (0.575-1.273) | 79 | 0.755 (0.535-1.066) | 293 | 0.728 (.605-0.876) |
| >300 min/wk | 6686 | 191 | 0.641 (0.452-0.908) | 221 | 0.556 (0.412-0.750) | 1087 | 0.674 (0.576-0.790) |
| **Moderate physical activity^c^** | | | |  |  |  |  |
| 0 min/wk | 2124 | 79 | Ref | 113 | Ref | 408 | Ref |
| 1-149 min/wk | 3382 | 103 | 0.925 (0.686-1.246) | 124 | 0.817 (0.630-1.059) | 563 | 0.858 (0.754-0.976) |
| 150-300 min/wk | 2226 | 62 | 0.857 (0.612-1.201) | 76 | 0.772 (0.575-1.037) | 341 | 0.800 (0.692-0.925) |
| >300 min/wk | 3274 | 108 | 0.912 (0.680-1.224) | 122 | 0.746 (0.575-0.967) | 590 | 0.881 (0.775-1.000) |
| **Walking^c^** |  |  |  |  |  |  |  |
| 0 min/wk | 1759 | 81 | Ref | 475 | Ref | 371 | Ref |
| 1-149 min/wk | 4852 | 153 | 0.759 (0.578-0.997) | 1134 | 0.722 (0.569-0.917) | 868 | 0.861 (0.761-0.974) |
| 150-300 min/wk | 2390 | 65 | 0.664 (0.477-0.926) | 464 | 0.599 (0.444-0.807) | 367 | 0.736 (0.636-0.852) |
| >300 min/wk | 2005 | 53 | 0.662 (0.466-0.940) | 397 | 0.597 (0.435-0.821 | 296 | 0.726 (0.622-0.847) |
| **Vigorous physical activity^c^** | | | |  |  |  |  |
| 0 min/wk | 6769 | 248 | Ref | 319 | Ref | 1266 | Ref |
| 1-74 min/wk | 1735 | 42 | 0.803 (0.576- 1.121) | 47 | 0.739 (0.541-1.009) | 262 | 0.879 (0.767-1.006) |
| 75-150 min/wk | 1111 | 26 | 0.777 (0.516-1.172) | 32 | 0.803 (0.555-1.163) | 170 | 0.861 (0.732-1.013) |
| >150 min/wk | 1390 | 36 | 0.863 (0.604-1.234) | 37 | 0.742 (0.525-1.050) | 204 | 0.850 (0.731-0.989) |
| **Moderate-to-vigorous physical activity (MVPA) /Sedentary behavior (SB)** | | | | |  |  |  |
| MVPA <150 min/wk, SB ≥7 hr/day | 330 | 13 | Ref | 15 | Ref | 62 | Ref |
| MVPA <150 min/wk, SB <7 hr/day | 2183 | 80 | 0.993 (0.532-1.852) | 115 | 1.074 (0.609-1.894) | 440 | 1.154 (0.873-1.527) |
| MVPA ≥150 min/wk, SB ≥7 hr/day | 1229 | 28 | 0.763 (0.363-1.600) | 33 | 0.718 (0.365-1.143) | 182 | 0.831 (0.602-1.147) |
| MVPA ≥150 min/wk, SB <7 hr/day | 7264 | 231 | 1.062 (0.505-2.231) | 272 | 0.906 (0.463-1.773) | 1218 | 0.948 (0.681-1.320) |

^a^ All models adjusted for age, education level, BMI, smoking, type 2 diabetes, family history of heart disease, ^b^ Model also adjusted for Sedentary Behaviour, ^c^ Model also adjusted for MVPA
