# Supplementary material for: Independent and joint associations of sedentary behaviour and physical activity with risk of recurrent cardiovascular events in 40,156 Australian adults with coronary heart disease
Source: Am J Prev Cardiol. 2025 Apr 17;22:100998. doi: 10.1016/j.ajpc.2025.100998 (PMC12041785; doi:10.1016/j.ajpc.2025.100998)
Supplement: Supplementary file 8 [file mmc8.docx]

**Supplementary 8.** Number of events and Hazard ratios (95% CI) for non-fatal cardiac events, total cardiac events, and major adverse cardiovascular events (MACE) by physical activity and sedentary behavior in females and males with coronary heart disease

|  |  | **Cardiac event^a^** | | **Cardiac event and death^a^** | | | | **MACE** | |
| --- | --- | --- | --- | --- | --- | --- | --- | --- | --- |
| **Females** | **n=15,278** | **No. of events** | **HRs (CI 95%)** | **No. of events** | **HRs (CI 95%)** | | | **No. of events** | **HRs (CI 95%)** |
| **Sedentary behavior^b^** | |  |  |  |  | | |  |  |
| ≥10.5 hr/day | 760 | 58 | Ref | 111 | Ref | | | 299 | Ref |
| 7-10.4 hr/day | 2688 | 187 | 0.798 (0.588-1.084) | 323 | 0.710 (0.567-0.889) | | | 988 | 0.809 (0.706-0.927) |
| 3.5-6.9 hr/day | 6238 | 412 | 0.693 (0.497-0.966) | 653 | 0.553 (0.430-0.709) | | | 2179 | 0.708 (0.607-0.824) |
| 0-3.4 hr/day | 5592 | 302 | 0.666 (0.446-0.995) | 415 | 0.436 (0.319-0.596) | | | 1600 | 0.688 (0.570-0.830) |
| **Moderate-to-vigorous physical activity^c^** | |  |  |  |  | | |  |  |
| 0 min/wk | 1261 | 112 | Ref | 203 | Ref | | | 541 | Ref |
| 1-149 min/wk | 3117 | 227 | 0.774 (0.616-0.973) | 400 | 0.793 (0.668-0.940) | | | 1146 | 0.759 (0.684-0.841) |
| 150-300 min/wk | 2379 | 154 | 0.775 (0.604-0.992) | 218 | 0.676 (0.556-0.822) | | | 784 | 0.720 (0.644-0.805) |
| >300 min/wk | 8516 | 466 | 0.668 (0.540-0.827) | 681 | 0.611 (0.519-0.720) | | | 2595 | 0.666 (0.605-0.732) |
| **Moderate physical activity^c^** | | | |  |  | | |  |  |
| 0 min/wk | 3517 | 281 | Ref | 491 | Ref | | | 1399 | Ref |
| 1-149 min/wk | 4196 | 245 | 0.845 (0.710-1.005) | 377 | 0.796 (0.694-0.912) | | | 1316 | 0.829 (0.768-0.894) |
| 150-300 min/wk | 2729 | 139 | 0.758 (0.617-0.932) | 203 | 0.701 (0.594-0.828) | | | 787 | 0.774 (0.709-0.846) |
| >300 min/wk | 4835 | 294 | 0.798 (0.675-0.942) | 431 | 0.715 (0.626-0.816) | | | 1564 | 0.781 (0.726-0.841) |
| **Walking^c^** |  |  |  |  |  | | |  |  |
| 0 min/wk | 2937 | 270 | Ref | 458 | Ref | | | 2371 | 0.822 (0.767-0.882) |
| 1-149 min/wk | 7141 | 427 | 0.705 (0.604-0.823) | 667 | 0.697 (0.618-0.786) | | | 821 | 0.751 (0.686-0.822) |
| 150-300 min/wk | 2861 | 153 | 0.691 (0.565-0.846) | 208 | 0.622 (0.527-0.735) | | | 671 | 0.741 (0.674-0.816) |
| >300 min/wk | 2335 | 109 | 0.590 (0.471-0.739) | 169 | 0.600 (0.502-0.717) | | |  |  |
| **Vigorous physical activity^c^** | | | |  |  | | |  |  |
| 0 min/wk | 7831 | 779 | Ref | 1261 | Ref | | | 3956 | Ref |
| 1-74 min/wk | 1535 | 94 | 1.005 (0.809-1.249) | 119 | 0.881 (0.728-1.065) | | | 510 | 0.962 (0.876-1.056) |
| 75-150 min/wk | 946 | 44 | 0.736 (0.541-1.000) | 58 | 0.709 (0.543 0.925) | | | 307 | 0.893 (0.794-1.005) |
| >150 min/wk | 1128 | 42 | 0.732 (0.535-1.002) | 64 | 0.830 (0.644-1.070) | | | 293 | 0.908 (0.805-1.024) |
| **Moderate-to-vigorous physical activity (MVPA)/Sedentary behavior (SB)** | | | | | |  |  | | |
| MVPA<150 min/wk & SB≥7 hr/day | 1201 | 108 | Ref | 226 | Ref | | | 535 | Ref |
| MVPA <150 min/wk & SB <7 hr/day | 2946 | 217 | 0.842 (0.661-1.072) | 356 | 0.681 (0.571-0.812) | | | 1071 | 0.815 (0.730-0.910) |
| MVPA≥150 min/wk & SB ≥ 7 hr/day | 2247 | 137 | 0.718 (0.534-0.964) | 208 | 0.556 (0.444-0.696) | | | 752 | 0.702 (0.614-0.803) |
| MVPA ≥150 min/wk & SB<7 hr/day | 8884 | 497 | 0.650 (0.473-0.893) | 712 | 0.465 (0.364-0.594) | | | 2708 | 0.633 (0.545-0.736) |
| **Men** | n= 24878 | **No. of events** | **HRs (CI 95%)** | **No. of events** | **HRs (CI 95%)** | | | **No. of events** | **HRs (CI 95%)** |
| **Sedentary behavior^b^** | |  |  |  |  | | |  |  |
| ≥10.5 hr/day | 1512 | 134 | Ref | 246 | Ref | | | 626 | Ref |
| 7-10.4 hr/day | 5196 | 493 | 0.997 (0.814-1.221) | 835 | 0.912 (0.783-1.061) | | | 2098 | 0.882 (0.802-0.971) |
| 3.5-6.9 hr/day | 10445 | 1065 | 1.036 (0.826-1.300) | 1661 | 0.853 (0.717-1.015) | | | 4146 | 0.831 (0.744-0.928) |
| 0-3.4 hr/day | 7725 | 609 | 0.939 (0.712-1.237) | 917 | 0.734 (0.592-0.911) | | | 2447 | 0.761 (0.664-0.873) |
| **Moderate-to-vigorous physical activity^c^** | | |  |  |  | | |  |  |
| 0 min/wk | 1561 | 155 | Ref | 316 | Ref | | | 693 | Ref |
| 1-149 min/wk | 4883 | 510 | 0.919 (0.767-1.101) | 886 | 0.817 (0.718-0.930) | | | 2053 | 0.810 (0.743-0.883) |
| 150-300 min/wk | 4213 | 417 | 0.872 (0.724-1.051) | 646 | 0.709 (0.619-0.813) | | | 1614 | 0.729 (0.667-0.798) |
| >300 min/wk | 14221 | 1219 | 0.755 (0.636-0.895) | 1811 | 0.597 (0.528-0.675) | | | 4957 | 0.659 (0.607-0.715) |
| **Moderate physical activity^c^** | |  |  |  |  | | |  |  |
| 0 min/wk | 5799 | 595 | Ref | 1049 | Ref | | | 2416 | Ref |
| 1-149 min/wk | 7977 | 704 | 0.865 (0.774-0.965) | 1076 | 0.788 (0.723-0.858) | | | 2892 | 0.852 (0.807-0.900) |
| 150-300 min/wk | 4276 | 403 | 0.908 (0.799-1.031) | 619 | 0.828 (0.749-0.916) | | | 1506 | 0.822 (0.770-0.877) |
| >300 min/wk | 6825 | 599 | 0.805 (0.718-0.903) | 915 | 0.713 (0.651-0.780) | | | 2503 | 0.810 (0.766-0.858) |
| **Walking^c^** |  |  |  |  |  | | |  |  |
| 0 min/wk | 4185 | 417 | Ref | 738 | Ref | | | 1794 | Ref |
| 1-149 min/wk | 11045 | 1066 | 0.969 (0.865-1.086) | 1697 | 0.915 (0.838-0.998) | | | 4182 | 0.869 (0.822-0.919) |
| 150-300 min/wk | 5165 | 427 | 0.820 (0.715-0.939) | 652 | 0.748 (0.673-0.832) | | | 1814 | 0.792 (0.741-0.846) |
| >300 min/wk | 4483 | 391 | 0.846 (0.736-0.972) | 572 | 0.745 (0.667-0.832) | | | 1527 | 0.757 (0.706-0.811) |
| **Vigorous physical activity^c^** | |  |  |  |  | | |  |  |
| 0 min/wk | 16069 | 1594 | Ref | 2684 | Ref | | | 6525 | Ref |
| 1-74 min/wk | 3814 | 319 | 0.905 (0.802-1.022) | 448 | 0.830 (0.750-0.918) | | | 1251 | 0.864 (0.813-0.918) |
| 75-150 min/wk | 2142 | 173 | 0.921 (0.786-1.079) | 243 | 0.857 (0.751-0.979) | | | 684 | 0.877 (0.810-0.949) |
| >150 min/wk | 2852 | 215 | 0.833 (0.721-0.962) | 284 | 0.732 (0.647-0.829) | | | 857 | 0.806 (0.750-0.866) |
| **Moderate-to-vigorous physical activity (MVPA)/Sedentary behavior (SB)** | | | | | | | |  |  |
| MVPA<150 min/wk & SB≥7 hr/day | 2027 | 208 | Ref | 432 | Ref | | | 970 | Ref |
| MVPA <150 min/wk & SB <7 hr/day | 4036 | 415 | 0.957 (0.803-1.139) | 706 | 0.770 (0.678-0.873) | | | 1627 | 0.803 (0.738-0.874) |
| MVPA≥150 min/wk & SB ≥ 7 hr/day | 4681 | 419 | 0.788 (0.647-0.959) | 649 | 0.619 (0.534-0.718) | | | 1754 | 0.671 (0.610-0.739) |
| MVPA ≥150 min/wk & SB<7 hr/day | 14134 | 1259 | 0.758 (0.608-0.945) | 1872 | 0.555 (0.470-0.656) | | | 4966 | 0.609 (0.545-0.680) |

^a^ All models adjusted for age, sex, education level, body mass index, smoking, type 2 diabetes, family history of heart disease, ^b^ Model also adjusted for Moderate-to-Vigorous Physical Activity, ^c^ Model also adjusted for Sedentary Behaviour, ^d^ lower risk (p < 0.05) compared to 1-140 minutes/week, ^e^ lower risk (p < 0.05) compared to 7-10.4 hours/day, ^f^ lower risk (p < 0.05) compared to MVPA <150 in/week, SB <7 hours/day
